# Real-time biomimetic Central Pattern Generators in an FPGA for hybrid experiments

**DOI:** 10.3389/fnins.2013.00215

**Published:** 2013-11-21

**Authors:** Matthieu Ambroise, Timothée Levi, Sébastien Joucla, Blaise Yvert, Sylvain Saïghi

**Affiliations:** ^1^Laboratoire IMS, UMR Centre National de la Recherche Scientifique, University of BordeauxTalence, France; ^2^Laboratoire INCIA (Institute for Cognitive and Integrative Neuroscience), UMR Centre National de la Recherche Scientifique, University of BordeauxTalence, France

**Keywords:** central pattern generator, biomimetic, neuron model, spiking neural networks, digital hardware, FPGA

## Abstract

This investigation of the leech heartbeat neural network system led to the development of a low resources, real-time, biomimetic digital hardware for use in hybrid experiments. The leech heartbeat neural network is one of the simplest central pattern generators (CPG). In biology, CPG provide the rhythmic bursts of spikes that form the basis for all muscle contraction orders (heartbeat) and locomotion (walking, running, etc.). The leech neural network system was previously investigated and this CPG formalized in the Hodgkin–Huxley neural model (HH), the most complex devised to date. However, the resources required for a neural model are proportional to its complexity. In response to this issue, this article describes a biomimetic implementation of a network of 240 CPGs in an FPGA (Field Programmable Gate Array), using a simple model (Izhikevich) and proposes a new synapse model: activity-dependent depression synapse. The network implementation architecture operates on a single computation core. This digital system works in real-time, requires few resources, and has the same bursting activity behavior as the complex model. The implementation of this CPG was initially validated by comparing it with a simulation of the complex model. Its activity was then matched with pharmacological data from the rat spinal cord activity. This digital system opens the way for future hybrid experiments and represents an important step toward hybridization of biological tissue and artificial neural networks. This CPG network is also likely to be useful for mimicking the locomotion activity of various animals and developing hybrid experiments for neuroprosthesis development.

## Introduction

Millions of people worldwide are affected by neurological disorders which disrupt connections between brain and body, causing paralysis or affecting cognitive capabilities. The number is likely to increase over the next few years and current assistive technology is still limited. In recent decades, extensive research has been devoted to Brain-Machine Interfaces (BMIs) and neuroprosthesis in general (Hochberg et al., 2006, 2012; Nicolelis and Lebedev, 2009), working toward effective treatment for these disabilities. The development of these devices has had and, hopefully, will continue to have a profound social impact on these patients' quality of life. These prostheses are designed on the basis of our knowledge of interactions with neuronal cell assemblies, taking into account the intrinsic spontaneous activity of neuronal networks and understanding how to stimulate them into a desired state or produce a specific behavior. The long-term goal of replacing damaged neural networks with artificial devices also requires the development of neural network models that match the recorded electrophysiological patterns and are capable of producing the correct stimulation patterns to restore the desired function. The hardware set-up used to interface the biological component is a Spiking Neural Network (SNN) system implementing biologically realistic neural network models, ranging from the electrophysiological properties of a single neuron to large-scale neural networks.

Our study describes the development of a neuromorphic hardware device containing a network of real-time biomimetic Central Pattern Generators (CPG). The main goal of this research is to create artificial CPGs that will be connected to *ex vivo* spinal cord of rats and guinea pigs, thus achieving one main objective of the Brainbow European project (Brainbow, 2012) toward hybridization. Hardware-based SNN systems were developed for hybrid experiments with biological neurons and the description of those pioneer platforms was reported in the literature (Jung et al., 2001; Le Masson et al., 2002; Vogelstein et al., 2006). The Brainbow project will go further by using a large-scale neural network instead of few neurons to substitute the functions of a biological sub-network. The final goal is the development of a new generation of neuro-prostheses capable to restore the lost communication between neuronal circuitries.

Locomotion is one of the most basic abilities of animals. Neurobiologists have established that locomotion results from the activity of half-center oscillators that provides alternating bursts. The first half-center oscillator was proposed by Brown (1914). Pools of interneurons control flexor and extensor motor neurons with reciprocal inhibitory connections. Most rhythmic movements are programmed by central pattern-generating networks consisting of neural oscillators (Marder and Bucher, 2001; Ijspeert, 2008). CPGs are neural networks capable of producing rhythmic patterned outputs without rhythmic sensory or central input. CPGs underlie the production of most rhythmic motor patterns and have been extensively studied as models of neural network function (Hooper, 2000). Half-center oscillators control swimming in xenopus, salamander (Ijspeert et al., 2007), and lamprey (Cohen et al., 1992), as well as leech heartbeat (Cymbalyuk et al., 2002), as described in numerous publications. One key article on modeling the leech heartbeat system is Hill et al. (2001), where the Hodgkin–Huxley formalism is used to reproduce the CPG.

The main novelty of this research was to implement the leech heartbeat system neural network with minimum resources while maintaining its biomimetic activity. Indeed, the final application is a hybrid experiment that requires spike detection, spike sorting, and micro-electrode stimulation. All of these modules are implemented in the same digital board. To achieve this, the Hill et al. (2001) model and results were reproduced using a simpler model (Izhikevich, 2004), implemented in an FPGA (Field Programmable Gate Array) board. This digital board made it possible to design a frugal, real-time network of several CPGs (in this case, a network of 240 CPGs implemented on a Spartan6 FPGA board). For instance, this CPG network is capable of mimicking the activity of a salamander, which requires 40 CPGs (Ijspeert, 2001), or developing hybrid experiments (Le Masson et al., 2002) for neuroprosthesis development (Brainbow, 2012).

The first part of this article describes the biological leech heartbeat system, based on one segmental CPG. The next section focuses on choosing a frugal neuron model to match the same biological behavior. The following section explains the topology of a single neuron and its implementation in the hardware, followed by its extension to a neuron computation core for increasing the size of the neural network. The next stage was to develop a new synaptic model reproducing activity-dependent depression phenomena to fit the biological activity of a leech heartbeat. The architecture of this digital system is then described in full, including the various blocks. Finally, the system was used to design a CPG network, validated by comparing our measurements with *ex vivo* rat spinal cord locomotion results following pharmacological stimulation.

## Materials and methods

### Description of the leech biological heartbeat system

**Figure 1:**
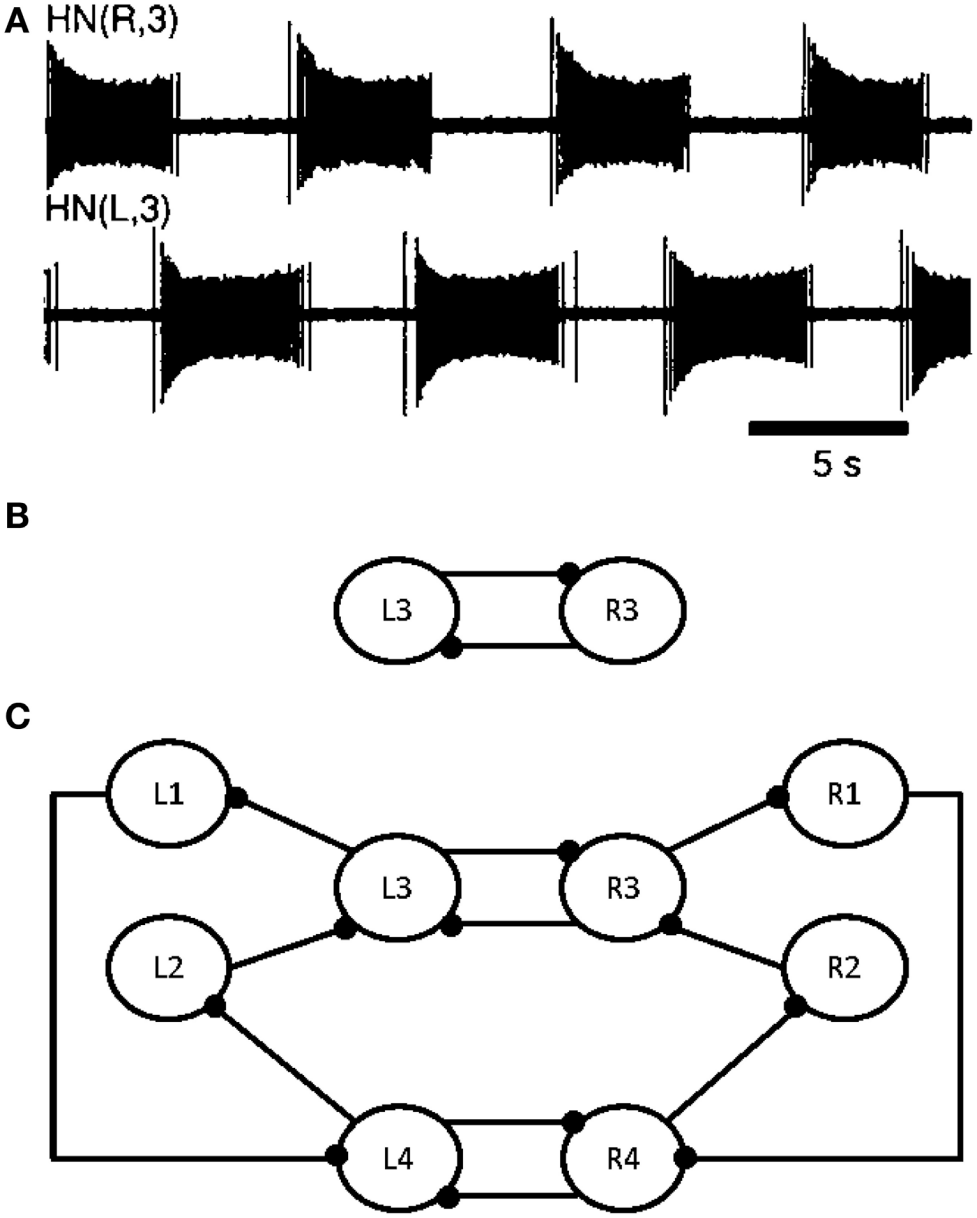
**Electrical activity of the leech heartbeat system and diagram of the CPG**. Neuron cell bodies are represented by circles. Axons and neurite processes are represented by lines. Inhibitory chemical synapses are represented by small filled dots. **(A)** Electrical activity of two heart interneurons recorded extracellularly from a chain of ganglia (Hill et al., 2001). **(B)** A diagram of the elemental oscillator in the leech heartbeat system. **(C)** A diagram of the segmental oscillator in the leech heartbeat system, including two elemental oscillators, L3/R3 and L4/R4, and two pairs of coordination neurons, L1/R1 and L2/R2.

All leech heartbeat studies agree that the CPG (Figure 1C) responsible for this activity (Figure 1A) requires few neurons, making it an ideal candidate system for elucidating the various biomechanisms governing CPG behavior.

Modeling studies indicate that the burst duration of a leech heart interneuron in an elemental oscillator is regulated by the interneuron itself and by the opposite interneuron (see L3 and R3 in Figure 1B) (Calabrese, 1995; Nadim et al., 1995; Olsen et al., 1995; Hill et al., 2001; Jezzini et al., 2004; Norris et al., 2007). Figure 1A shows the electrical activity in the leech heartbeat system from extracellular recordings. The pair of neurons is known as an elemental oscillator (Figure 1B), i.e., the smallest unit that capable of producing robust oscillations under normal conditions. These neurons oscillate in alternation with a period of about 10–12 s (Krahl and Zerbst-Boroffka, 1983; Calabrese et al., 1989; Olsen and Calabrese, 1996) demonstrated that the synaptic connections among interneurons and from interneurons to motor neurons were inhibitory. The synaptic interaction between reciprocally inhibitory heart interneurons consists of a graded component in addition to spike-mediated synaptic transmissions (Angstadt and Calabrese, 1991). This kind of synapse is really difficult to implement in hardware as it contains sigmoid functions, differential equations, memory of last spikes, and so on. A description of our synapse model reproducing the same behavior is included below.

Nadim et al. (1995) and Olsen et al. (1995) developed a biophysical model of a pair of reciprocally inhibitory interneurons in the leech heartbeat system. This model included synaptic ionic currents based on voltage-clamp data. Synaptic transmissions between the interneurons consist of spike-mediated and graded synaptic currents. The Hill et al. (2001) model was derived from a previous two-cell, elemental oscillator model (Nadim et al., 1995) by incorporating intrinsic and synaptic current modifications based on the results of a realistic waveform voltage-clamp study (Olsen and Calabrese, 1996). This new, segmental oscillator model behaves more similarly to biological systems. Figure 1C shows a model of the system. The real-time digital segmental oscillator model design will be based on this architecture. The next part will describe the system modeling the leech heartbeat with the goal of implementing it in hardware. The leech heartbeat CPG was chosen for the long duration of the burst.

### System modeling for hardware implementation

#### State of art

Some previous studies used silicon neurons (Indiveri et al., 2011) to simulate the leech heartbeat system (Simoni et al., 2004; Simoni and DeWeerth, 2007). Sorensen et al. (2004) created a hybrid system of a heart interneuron and a silicon neuron. The silicon neuron provides real-time operation and implements a version of the Hodgkin–Huxley formalism (Hodgkin and Huxley, 1952). However, due to the complexity of the model, it was only possible to use a small number of silicon neurons and, therefore, only one CPG. This study describes the same results using a large CPG network (240 CPGs on a Spartan6 FPGA board), in preparation for future hybrid experiments with different CPGs. For instance, in the salamander model (Ijspeert, 2001), the body CPG consists of 40 interconnected segmental networks.

When a silicon neuron and heart interneuron are connected with reciprocal inhibitory synapses of appropriate strength, they form a hybrid elemental oscillator that produces oscillations remarkably similar to those seen in the living system. Olypher et al. (2006) described the control of burst duration in heart interneurons using a hybrid system, where a living, pharmacologically-isolated, heart interneuron was connected with artificial synapses to a model heart interneuron running in real-time (software). Using an FPGA board will make it possible to operate in real time using a large number of neurons, together with customized systems for various applications (hybrid experiments).

A few studies (Torres-Huitzil and Girau, 2008; Rice et al., 2009; Serrano-Gotarredona et al., 2009; Barron-Zambrano et al., 2010; Barron-Zambrano and Torres-Huitzil, 2013) reported on CPG in FPGA for robotic applications. These studies used simple neuron-models and were more bio-inspired than biomimetic. Guerrero-Riberas et al. (2006) implemented a network of LIF neurons with synapses and plasticity, but not in biological time, so it was impossible to perform hybrid experiments. While multi-legged robots need CPG to move or coordinate their movements, they implement an Amari–Hopfield CPG (Amari, 1972) or basic CPGs (Van Der Pol, 1928), modeled as non-linear oscillators. Those models provide sinusoidal oscillations that are not biorealistic. The ultimate goal of these studies is to create a robot that mimics biological behavior but these systems cannot be used for hybrid experiments. Analog hardware has also been implemented (Linares-Barranco et al., 1993; Still and Tilden, 1998; Lewis et al., 2001; Nakada, 2003; Still et al., 2006; Lee et al., 2007; Wijekoon and Dudek, 2008). However, it is very difficult to tune analog circuits due to parameter mismatch. For these works, they either design bio-inspired oscillators for creating CPG or implement few biomimetic neurons.

#### Choice and presentation of the izhikevich model

In designing a SNN, the first step is the choice of a biologically realistic model. Indeed, a mathematical model based differential equations is capable of reproducing a behavior quite similar to that of a biological cell. The choice of model was based on two criteria: the family of neurons able to be reproduced and the number of equations. These criteria were used to compare several models, including the Leaky Integrate and Fire model (LIF) (Indiveri, 2007), the Hodgkin–Huxley model (HH), and the Izhikevich model (IZH).

Hill et al. (2001) used the HH to reproduce the leech heartbeat system with eight neurons (Figure 1C). From the equations defined in this paper, it was established that the eight neurons in the heartbeat leech behaved like regular spiking ones (RS). Indeed, this model was composed of nine voltage-dependent currents with different calcium conductances.

The HH model reproduces all types of neurons with good accuracy (spike timing and shape). Its main drawbacks are the large number of parameters and the equations required. In the heartbeat network, the main focus is on excitatory neurons, like RS. The HH model required 32 parameters for an RS and 26 for a fast-spiking neuron (FS) (Grassia et al., 2011). Furthermore, simulating an RS neuron required four ionic channels (dynamics of potassium and sodium ions, leak current, and slow potassium). In contrast, LIF only involves two equations but is only capable of simulating a few types of neurons.

The IZH represents a good solution, as it is based on two equations and is capable of reproducing many different families of neurons by changing four parameters. Furthermore, according to Izhikevich (2004), this model is resource-frugal, a key advantage when the aim is to design a large CPG network embedded in the same board as other modules required for hybrid experiments (spike detection, spike sorting, stimulation, etc.).

The IZH model depends on four parameters, which make it possible to reproduce the spiking and bursting behavior of specific types of cortical neurons. From a mathematical standpoint, the model is described by a two-dimensional system of ordinary differential equations (Izhikevich, 2003):

(1) $$\frac{dv}{dt}=0.04v^{2}+5v+140-u+I_{\text{Izh}}$$

(2) $$\frac{du}{dt}=a(bv-u)$$

with the after-spike resetting conditions:

(3) $$\text{if }v\geq 30\text{mV}\Rightarrow \{\begin{array}{l} v\leftarrow c\\ u\leftarrow u+d \end{array}$$

In equation (3), *v* is the membrane potential of the neuron, *u* is a membrane recovery variable, which takes into account the activation of potassium and inactivation of sodium channels, and *I*_Izh_ describes the input current from other neurons.

The IZH model was chosen to emulate the behavior of the excitatory cells for its simplicity and its capacity to implement various families of neurons. The next step was to determine the network system topology. The next section describes the design of one neuron and its extension to a neuron computation core, then the different synapse models implemented, and, finally, the topology of the network.

### System topology

#### Topology of one neuron core: architecture and implementation

In order to make the Izhikevich neural network more biomimetic, the *I*_Izh_ current from equation (1) was split into three: *I*_bias_, *I*_exc_, and *I*_inh_. *I*_bias_ is the biasing current, *I*_exc_ is the positive contribution due to excitatory synapses, and *I*_inh_ is the negative contribution of inhibitory synapses. Those currents will be detailed in Synapse Model. As suggested in Cassidy and Andreou (2008), equation (1) was multiplied by 0.78125 to make it easier to implement on a digital board. These modifications gave (4), where the *u* coefficient is still 1 thanks to *I*_bias_ current.

(4) $$\begin{array}{l} \frac{dv}{dt}=1/32v^{2}+4v+109.375-u+I_{\text{bias}}+I_{\text{exc}}+I_{\text{inh}}\\ \frac{du}{dt}=a\cdot (bv-u) \end{array}$$

Moreover, $\frac{dv}{dt}=\frac{v\left[n+1\right]-v\left[n\right]}{\Delta t}$ and, as the time step of the IZH model is equal to one millisecond (Δ*t* = 1):

(5) v[n+1]=132v[n]2+5v[n]+109.375−u[n]+Ibias[n]+Iexc[n]+Iinh[n]u[n+1]=u[n]+a.(b.v[n]−u[n])

One neuron was implemented on the FPGA board according to these equations and specifications. This neuron was then extended into a neuron computation core that updated the *u* and *v* values of all neurons in the network. Consequently, the neuron implementation became a neuron computation core. For instance, around 2000 independent neurons could be implemented on our digital board. In this system, the type of neuron is defined by the four Izhikevich parameters: a, b, c, and d from equations (2) and (3). Moreover, the state of a neuron is defined by values u and v, and the three current values. Those 9 values were saved in a RAM for use in the next millisecond in the step computation. By extension, the same process can be used for every neuron in the network.

**Figure 2:**
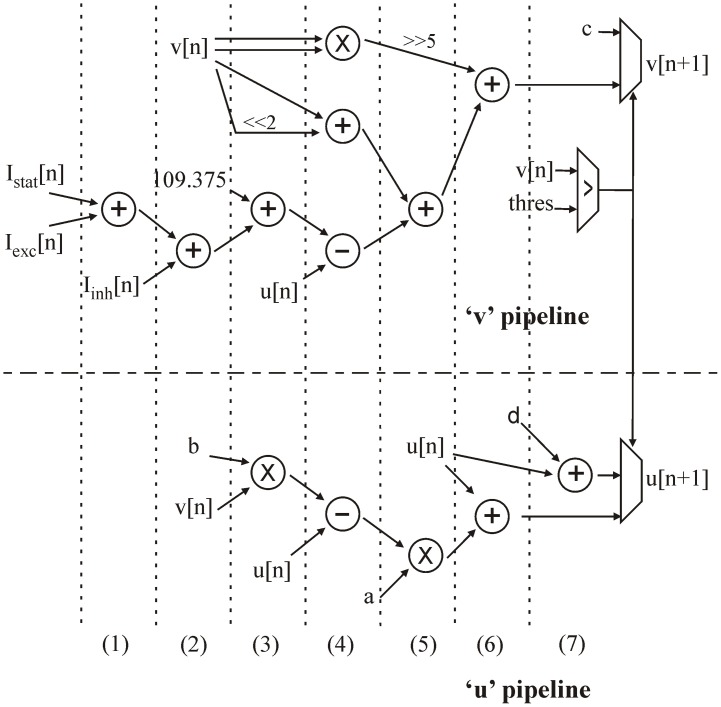
**Architecture of the “u” and “v” pipelines in the neural computation core**. The computation cycles are separated by dotted lines.

Each *u* and *v* computation step is run in parallel, using two pipelines based on the architecture presented in [9]. The topology is presented in Figure 2. All parameters from equations (2), (3), and (4), as well as the *u* and *v* values used in the computation are synchronized in one cycle before going through the pipelines (not shown in Figure 2).

To resume, each neuron is represented by one “v” and “u” value, four Izhikevich coefficients (a, b, c, and d), and three currents (*I*_bias_, *I*_exc_, and *I*_inh_).

*I*_exc_, *I*_inh_, and *I*_bias_ are added in two cycles at the beginning of the “*v*” pipeline, while the “*u*” pipeline is still inactive (steps 1 and 2). The current sum is added to the constant *109.375* and at the same time as the first multiplication (step 3). By multiplexing operands, the same multiplier is used for the following multiplications in different computation cycles. In step 4, *v*^2^ is obtained by another multiplication. A simple two-bit shift makes it possible to obtain *4v* and add it to *v*. At the same time, *u* is used in two subtractions. Step 5 consists of a 5-bit shift to obtain *(1/32)v*^2^, an addition, and the last multiplication. In step 6, the computation of both *u* and *v* is completed. In the next step, the *v* value is tested against the threshold to determine whether the neuron has emitted a spike or not. This test gives the next *u* and *v* values for this neuron to be stored in the RAM.

An RS neuron with *a* = 0.002, *b* = 0.2, *c* = −65, and *d* = 8 was used to implement the CPG.

Once the neuron computation core was implemented, the synaptic model was chosen and implemented.

#### Synapse model

A network is defined by a group of neurons and a group of synapses. Once the neuron model had been chosen, it was obviously necessary to choose a synapse model. Like the neuron model, this model had to be biomimetic but frugal in its use of resources. In biology, synapses are described as links between neurons that transmit different types of synaptic currents to each other to either excite or inhibit neuron activity. In our implementation, a synaptic weight (*W*_syn_) was added to the synaptic current. When *W*_syn_ was positive, it was added to *I*_exc_ (excitatory synaptic current) and when *W*_syn_ was negative, it was added to *I*_inh_ (inhibitory synaptic current).

Thanks to AMPA and GABA effects, all synaptic current excitations or inhibitions, respectively decay exponentially (Ben-Ari et al., 1997). AMPA is an excitatory neurotransmitter that depolarizes the neuron membrane whereas GABA is an inhibitory neurotransmitter with a hyperpolarizing effect. Depolarization or hyperpolarization are represented by a positive or negative contribution on the synaptic current.

The synaptic current *I*_syn_ was implemented with a time constant τ_syn_ for the exponential decay, as follows:

(6) $$I_{\text{syn}}(t)=-\tau_{\text{syn}}\cdot I_{\text{syn}}^{\prime }(t)=-\tau_{\text{syn}}.\frac{I_{\text{syn}}(t+T)-I_{\text{syn}}(t)}{T}$$

(7) $$I_{\text{syn}}(t+T)=\left(1-\frac{T}{\tau_{\text{syn}}}\right)\cdot I_{\text{syn}}(t)$$

When computation step T equals one millisecond and τ_syn_ is in ms:

(8) $$I_{\text{syn}}(t+1)=\left(1-\frac{1}{\tau_{\text{syn}}}\right)\cdot I_{\text{syn}}(t)$$

(9) $$I_{\text{syn}}\left[n+1\right]=I_{\text{syn}}\left[n\right]-\frac{1}{\tau_{\text{syn}}}\cdot I_{\text{syn}}\left[n\right]$$

Adding the synaptic weight to the synaptic current, the new equation is:

(10) $$I_{\text{syn}}\left[n+1\right]=I_{\text{syn}}\left[n\right]-\frac{1}{\tau_{\text{syn}}}\cdot I_{\text{syn}}\left[n\right]+W_{\text{syn}}\left[n\right]$$

The synaptic computation core implementation is based on the same principle as the neuron computation core. However, this model is not adequate to fit biological data. It was, therefore, decided to implement an activity-dependent depression, where the new synaptic weight, *W*_s_, was dependent on *W*_syn_.

#### Activity-dependent depression

As the synaptic behavior described in Hill et al. (2001) requires too many resources to be implemented on FPGA, the method chosen to fit overall biological behavior was activity-dependent depression (Tabak et al., 2000). Activity-dependent depression of synapses is another biological phenomenon consisting of reducing a synaptic weight after a spike. In biology, each synapse contribution is provided by a synaptic vesicle. These vesicles contain ions that empty out at each spike and then regenerate, following an exponential rule. According to Matsuoka (1987), four methods provide a stable rhythm within a network (regulation of stimulus intensity, change in input, alteration of stimuli, and change in synaptic weight). The phenomenon, known as activity-dependent depression changes the synaptic weight depending on the activity of the network.

This phenomenon has been reported in neurobiology literature but no model had been devised. This paper proposes a model of this activity-dependent depression that was implemented in digital hardware to improving our CPG network.

As previously explained, each time a neuron emits a spike; the synapse adds a synaptic weight (*W*_syn_) to the synaptic current. At the same time, the factor (δ_syn_) indicating the level of depression on a synaptic weight increases. Furthermore, δ_syn_ regulates *W*_syn_. The value of δ_syn_ is between 0 and 1. Consequently, when δ_syn_ equals zero there is no depression on *W*_syn_ and when δ_syn_ equals one there is maximum depression on *W*_syn_ and the synapse is exhausted.

*W*_s_ was used instead of *W*_syn_ as the synaptic weight for each synapse. Then, according to the activity-dependent depression effect, when there is a spike, *W*_s_ is added to the synaptic current:

(11) $$\begin{array}{l} W_{\text{s}}\left[n\right]=W_{\text{syn}}-\delta_{\text{syn}}\left[n\right]\cdot W_{\text{syn}} \end{array}$$

The other effect of activity-dependent depression is to increase δ_syn_ after each spike, thanks to the percentage dissipation (*P*).

(12) $$\delta_{\text{syn}}\left[n+1\right]=\delta_{\text{syn}}\left[n\right]+P\cdot (1-\delta_{\text{syn}}\left[n\right])$$

The regeneration or reloading of synaptic vesicles is represented by δ_syn_ decreasing to zero. Thus, δ_syn_ decays exponentially when no spike is emitted. So, using the method described in Synapse Model:

(13) $$\delta_{\text{syn}}\left[n+1\right]=\delta_{\text{syn}}\left[n\right]-\frac{1}{\tau_{\text{reg}}}\cdot \delta_{\text{syn}}\left[n\right]$$

To summarize, all synapses are now represented by (12), (13), (14) and:

(14) $$I_{\text{syn}}\left[n+1\right]=I_{\text{syn}}\left[n\right]-\frac{1}{\tau_{\text{syn}}}\cdot I_{\text{syn}}\left[n\right]+W_{\text{s}}\left[n\right]$$

The main parameters are: synaptic weight, *W*_syn_; level of depression, δ_syn_; and percentage dissipation, *P*. All these parameters are stored in the RAM on the digital board. Furthermore, this computation required greater precision due to the sensitivity of the parameters. The 26-bit signed fixed representation chosen had 1-bit for the sign, 9-bits for the whole numbers, and 16 for the decimals.

Once the neuron and synapse models had been designed, it was possible to develop the neural network topology.

#### Network topology

**Figure 3:**
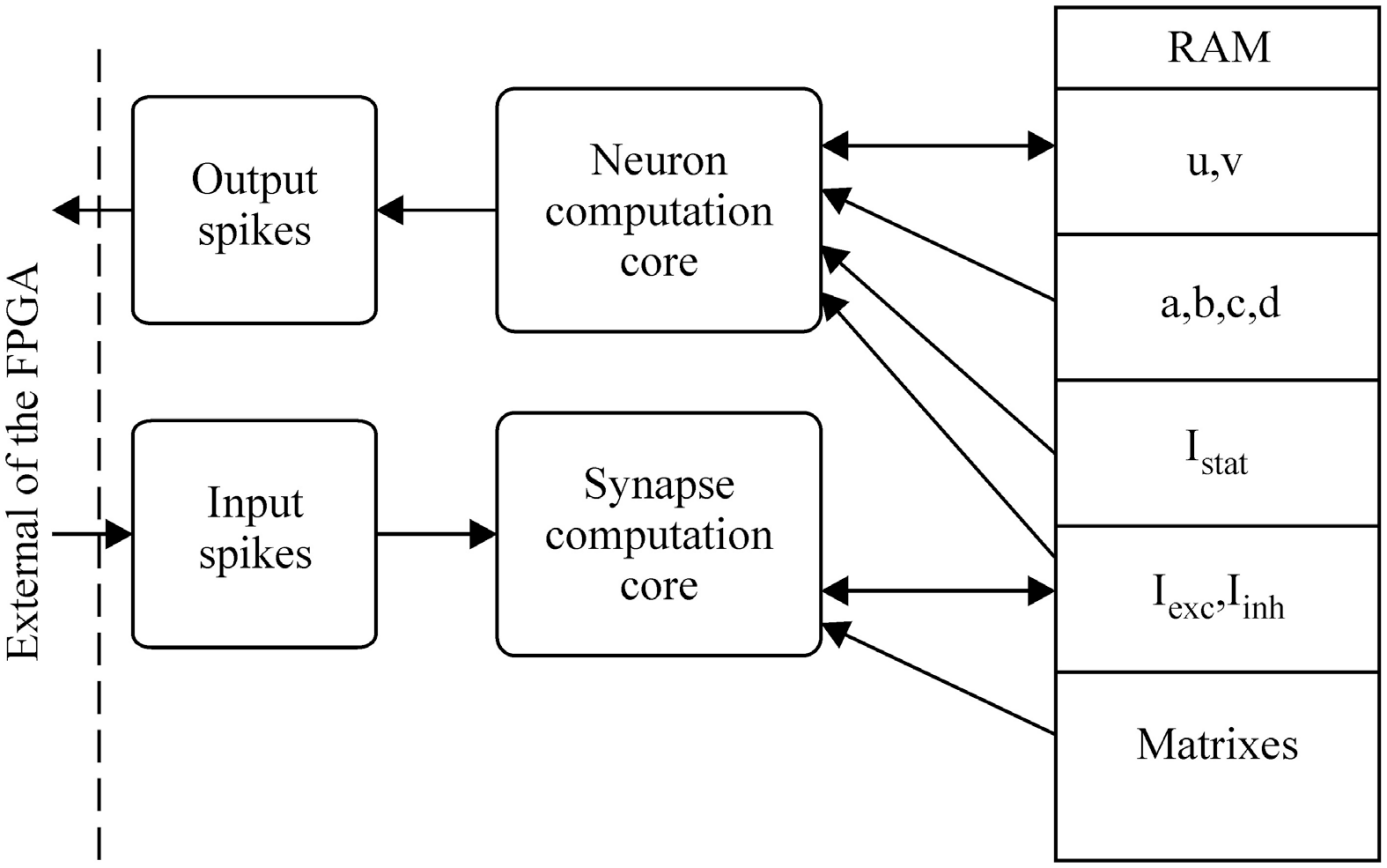
**Global architecture of the spiking neural network**.

***Three elementary blocks.*** The architecture was based on three main blocks: the neuron implemented (or neuron computation core), a synapse, and the RAM. The connectivity between those blocks is shown in Figure 3.

So far, the neuron computation core can update the state (“u” and “v” variables) of each neuron. In the digital network, the role of the synapse is to update all synaptic currents and weights related to the activity of all neurons, so the synapse block exhibits two behaviors (spiking or not). These two behaviors are summarized in Table 1.

**Table 1:** **Description of the equations for synaptic currents and activity-dependent depression**.

**When a spike is emitted**	**When no spike is emitted**
Synaptic current	Synaptic current
*I*_exc_ [*n* + 1] = *I*_exc_ [*n*] + *W_s_* [*n*] or *I*_inh_ [*n* + 1] = *I*_inh_ [*n*] + *W_s_* [*n*]	*I*_exc_ [*n* + 1] = *I*_exc_[*n*] and *I*_inh_ [*n* + 1] = *I*_inh_ [*n*]
Activity-dependent depression	Activity-dependent depression
δ_syn_ [*n* + 1] = δ_syn_ [*n*] + *P*(1 − δ_syn_ [*n*])	$\delta_{\text{syn}}\left[n+1\right]=\delta_{\text{syn}}\left[n\right]-\frac{\delta_{\text{syn}}\left[n\right]}{\tau_{\text{reg}}}$

The IZH model has a time step of one millisecond, so the other computation was synchronized with this time step. The new values of u and v, the exponential decay of *I*_syn_, and the new values of each synaptic current are computed in the same millisecond.

Moreover, a biological neural network is composed of *Nn* neurons and *Ns* synapses. To define which neuron is connected to which and with which kind of synapse (excitatory or inhibitory), the network is described using two matrixes: connectivity and synaptic weight (see Figure 3). To save RAM, both matrixes are implemented as sparse matrixes with *Nn* lines. The *i*th line in the connectivity matrix corresponds to the connectivity of presynaptic neuron *N_i_* to the other neurons. The synapses are identified by the postsynaptic neuron addresses. For example, the connection to neuron N_*j*_ is identified by the number *j* on the *i*th line. In the worst case, each neuron is connected to itself and all the others, giving *Nn* columns. Each matrix line ends with a virtual neuron (address *Nn* + 1). This implementation is not optimum for the worst case, but the gain is significant for biologically plausible networks, where the total number of synapses is at least four times smaller. Marom and Shahaf (2002) and Garofalo et al. (2009) estimated the average connectivity level of neural networks at their mature phase each neuron is mono-synaptically connected to 10–30% of all the other neurons.

**Figure 4:**
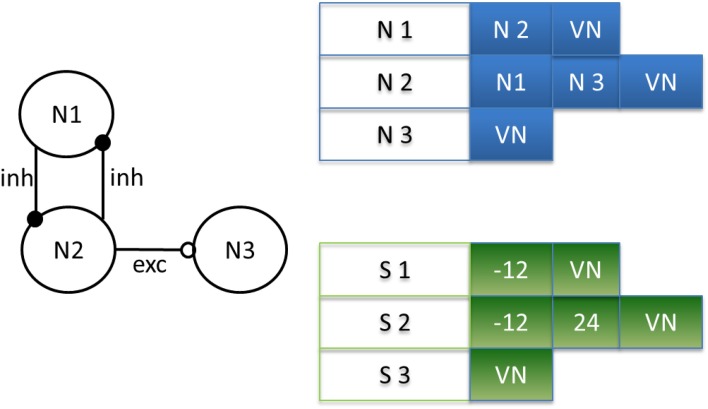
**Example of matrix design depending on the neural network**. Neuron 2 (N2) is connected by an inhibitory synapse to neuron 1 (N1) and by an excitatory synapse to neuron 3 (N3). Then, on line 2 in the connectivity matrix, N2 is connected to N1, N3, and a virtual neuron (VN), indicating the end of the connection. In the synaptic weight matrix, the synapses for neuron 2 (S2) have a negative weight for the inhibitory synapse and a positive weight for the excitatory synapse. Note the correspondence of its position in both matrixes.

There is a direct link between the matrixes: the synaptic weight matrix is the same size as the connectivity matrix, i.e., the same number of lines and columns, with the virtual neurons in the same position (Figure 4). The connectivity between two neurons described by the coordinates (*k*, *l*) in the connectivity matrix has the weight shown in box (*k*, *l*) in the synaptic weight matrix. A third matrix based on the same principle completes the system: the percentage efficiency matrix, which gives the percentage dissipation, *P*, of each synapse in a network, as defined in the previous section on activity-dependent depression. We will describe now the state machine of the neural network.

***Network machine states.*** The synaptic current is computed in three successive steps:

– EXT state: for closed-loop experiments, we implement this state in which external feedback can interact with the artificial neural network. This first state consists of using the synaptic block to update the synaptic current. In this case, presynaptic spikes are external events (see Figure 3), such as stimulation from biological neurons in the case of neuroprosthesis. This state makes it possible to stimulate each neuron.
– NEUR state: during this step, the neuron membrane (“*u*” and “*v*” from Figure 2) and all exponential decay values are computed in parallel.
– SYN state: the last step consists of updating the synaptic current to reflect the presynaptic spikes computed in the NEUR state. These updated current values are used in the EXT state during the next cycle.

The EXT, NEUR, and SYN states must be completed within a one millisecond time step. If the computation of all three states is completed in less than 1 ms, an IDLE state is implemented until the end of the cycle. Moreover, the blocks (neuron computation core and synapse computation core) described in Figure 3 are multiplexed in time to reduce the implementation area in large-scale neural networks.

Our architecture has two main limits: the number of available cycles (Nc) in one millisecond and the size of the RAM used to save all parameters. Two equations derived from these limits determine the maximum size of the implementable neural network, in terms of number of neuron (Nn) and synapses (Ns).

In the EXT state, all synaptic currents are updated in 10 cycles for each neuron, i.e., 10· Nn cycles. Each neuron requires 11 cycles to compute the NEUR state, i.e., 11· Nn cycles. The synaptic current update during state SYN requires 10 cycles per synapse, i.e., 10· Ns. Figure 2 describes 7 cycles for the neuron computation core, but 4 more cycles are required to read and save the various parameters in the RAM.

This leads to the following equation for computing the maximum number of neurons that may be implemented, depending on the number of cycles available:

(15) 10·Nn+11·Nn+10·Ns≤Nc

Having built all the component parts of this real-time, biomimetic digital system, it was possible to validate it by several experiments, presented in the following section.

## Results

A CPG is defined by the number of neurons and the families of neurons and synapses. The leech heartbeat neural network was simulated by an appropriate CPG configuration.

Hill et al. (2001) presented an elemental oscillator, based on two excitatory neurons linked by inhibitory synapses. A segmental oscillator may consist of 4–10 neurons. A two-neuron network (elemental oscillator from Figure 1B) was chosen to validate our topology, followed by an eight-neuron neural network (segmental oscillator from Figure 1C). The activity of our system was then compared with that of an *ex vivo* rat spinal cord, stimulated with pharmacological solutions. It was also demonstrated that the period of bursting activity could be modified depending on one parameter. This will be useful in future closed-loop hybrid experiments.

Biological CPGs provide specifications concerning their behavior. Indeed, their activity is characterized by periodic long bursts (lasting many seconds). Each burst begins by a quick rise in spike frequency to a maximum and ends with a low final spike frequency.

### Comparison of biological/digital elemental oscillator

The first example of a CPG was the elemental oscillator (with only two neurons). To reproduce activity accurately, it was necessary to obtain the following values: τ_ampa_ (time constant of the inhibitory synaptic current exponential decay), τ_reg_ (time constant of the recovery of synaptic vesicles), and *P* (percentage dissipation). These values will be the same for each synapse. The following values were chosen to match biological behavior: τ_current_ = 100 ms and τ_reg_ = 4444 ms (so 1/τ_current_ = 0.01 and 1/τ_reg_ = 0.0002). The *I*_bias_ current was equal to 8 for both neurons. The synaptic weights are −5.1 and the percentages of dissipation are 1.49.

**Figure 5:**
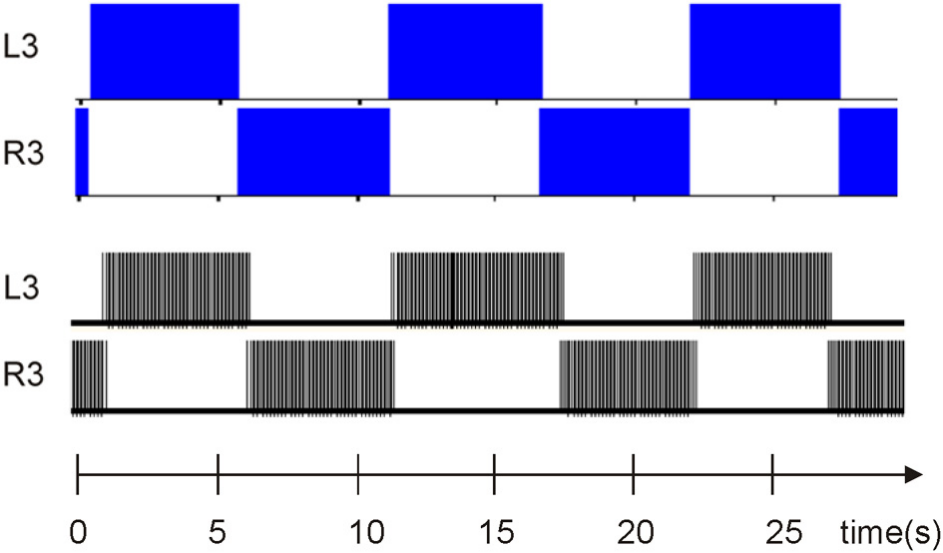
**Comparison between elemental oscillator (Figure 1B) bursting activity in the complex model simulated by scilab, as described in Hill et al. (2001) and the elemental oscillator presented above thanks to a logic analyzer**. The time scale is the same.

This model was validated by comparing its implementation with the complex model in Hill et al. (2001) (see Figure 5).

In this case, the activity of one neuron inhibits the second neuron. Due to activity-dependent depression and the GABA effect, the inhibition ends and lets the second neuron fire again. In both cases (biological modeling system and digital system), the bursting activity was similar in terms of period and duty cycle, thus validating the simplified elemental oscillator with the complex one. The next step was to validate the segmental oscillator and compare its implementation with biological data.

### Comparison of biological/digital segmental oscillator

Keeping the time constant, the biological behavior of the eight-neuron network was duplicated using the following parameters. This time, an eight-neuron CPG was implemented using the same values for τ_current_ and τ_reg_ than as those used for the elemental oscillator. The use of 8 neurons made it possible to maintain the period without variation (see Table 2) by slowing down the two pairs of oscillators with coordination neurons (De Schutter, 2000).

**Table 2:** **Comparison of burst characteristics in the two digital implementations and the biological system**.

	**Biological system (Hill et al., 2001)**	**Elemental oscillator (digital)**	**Segmental oscillator (digital)**
Mean period	10–12 s	12.6 ± 1.4 s	11.2 ± 1 s
Mean duty cycle	57.2 ± 2.9%	54.7 ± 6%	46.1 ± 6%
Mean spike frequency	11.9 ± 2.1 Hz	12.1 ± 1 Hz	11.2 ± 1 Hz
Initial spike frequency	4.3 ± 0.7 Hz	8.5 ± 0.2 Hz	8.6 ± 0.4 Hz
Peak spike frequency	17.5 ± 3.2 Hz	13 ± 0 Hz	12.5 ± 0 Hz
Final spike frequency	5.8 ± 1.0 Hz	8.1 ± 0.2 Hz	9.3 ± 3 Hz

**Figure 6:**
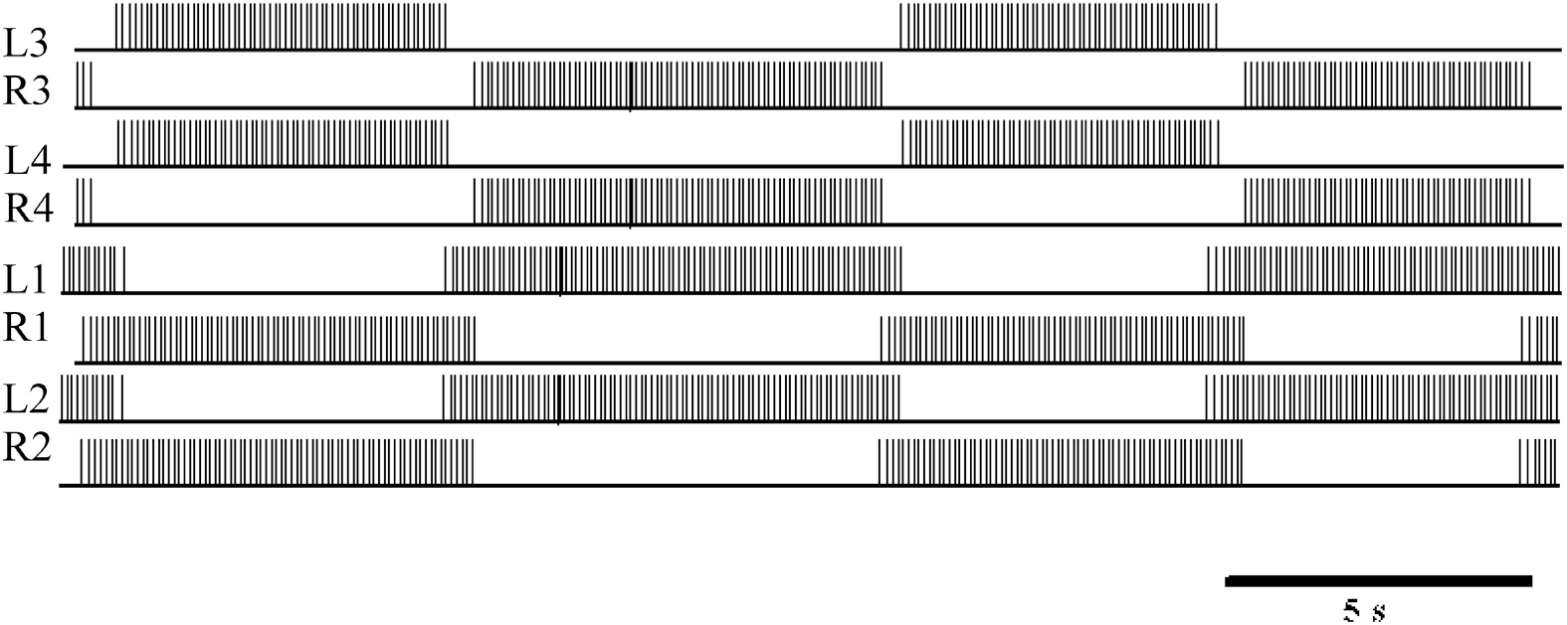
**Logic analyzer measurements of the digital eight-neuron CPG**. L3 and R3 show the activity of the first oscillator. L4 and R4 show the activity of the second oscillator. L1/R1 and L2/R2 are the coordination neurons.

In Figures 1C, 6, L3/R3 and L4/R4 correspond to the two elemental oscillators and are coupled to the L1/R1 and L2/R2 coordination neurons. The connectivity between each neuron is following Figure 1C. The synaptic weights are −7 and the percentage of dissipation is 2.65.

The mean period, duty cycle, and variations in spike frequency depending on their position in the burst were measured to quantify the overlap of bursting activity (Table 2). The mean period of this digital implementation was similar to biological values. Note that the segmental oscillator exhibited less variation than the elemental system, thanks to its coordination neurons.

Also, in general, the spike frequency of our implementation was similar to that of the biological system. Due to our synapse model, the frequency reached a maximum in each spike burst but remained on a plateau instead of decreasing to the minimum frequency immediately. In the biological system, the behavior described is due to the enhancement and attenuation of variations in conductance. However, the IZH model does not include conductance, so it cannot be as biomimetic as the HH model. This highlights a weak point of the implementation presented here, but even the HH model, Hill et al. (2001) was unable to mimic this biological variation in spike frequency in a single burst. One discrepancy between the model and the biological system is that the initial and final spike frequencies of a burst were consistently lower in the biological system. In both implementations, the most inconvenient drawback was the variation in the duty cycle, explained by the stability of the IZH model. One perspective of this work to ensure stability is described in the discussion section.

These experiments validated the implementation of our elemental and segmental oscillators. This table also confirms that designing a biomimetic system was a good choice. Indeed, the variations of the duty cycle and the period for the bursting activity could not be reproduced by bio-inspired oscillators. The next step was to identify one parameter that would modify the bursting activity period, which would be useful in closed-loop applications.

### Variation in the mean period depending on one parameter

A CPG is defined here by the number of neurons and the type synapses involved, the static currents of each neuron, the percentage dissipation, and the synaptic efficiency time constant.

Changing the synaptic efficiency time constant τ_reg_ modifies the period of each spike burst (Table 3). The variation in τ_reg_ affects the period and duration of each burst, as well as the duty cycle and the variability of these parameters: the greater the value of 1/τ_reg_, the longer the mean period of bursting activity.

**Table 3:** **Variation in the mean period depending on the τ_reg_ parameter**.

**1/τ_reg_(ms^−1^)**	**Mean period (s)**
0.09	4.4 ± 1.6
0.15	7.2 ± 1.2
0.20	11.2 ± 1
0.22	12.9 ± 1.1

The possibility of modifying the period using a single parameter is very useful and was applied in a closed-loop hybrid experiment concerning locomotion behaviors.

### FPGA resources

Originally, a CPG consisted of 8 neurons and 12 synapses but 2 additional synapses per CPG were required to create a network of CPG, by connecting CPG to another one. Thus, each CPG consisted of 8 neurons and 14 synapses. In terms of cycles and available memory, this implementation was capable of running 240 CPGs on a Spartan 6 digital board [see equation (15) and Table 4]. The power consumption of one CPG is 8 mW and for CPGs is 20 mW. We could reduce it in the future by designing a custom ASIC. For neuroprosthesis application, the power consumption should be lower than 80 mW/cm2 chronic heat dissipation level considered to prevent tissue damage (Zumsteg et al., 2005).

**Table 4:** **Resources required for one CPG on a Spartan 6 digital board**.

**Resources**	**Total available**	**Used for one CPG**	**Used for 240 CPGs**
Slice FF's	184304	1,093 (0.6%)	1,459 (0.8%)
Slice LUT	92152	1,037 (1.2%)	1,756 (1.9%)
DSP48A1	180	10 (5.6%)	10 (5.6%)
RAMB16BWER	268	1	42 (756 kb)
Total RAM	4824 kb	9 kb (0.2%)	765 kb (16%)

### Comparison with *ex-vivo* rat spinal cord results using pharmacological stimulation

The final validation of this system consisted of comparing the CPG output with *ex vivo* physiological data obtained from the spinal cord of newborn rat [postnatal day (P)1–2]. Bursting locomotor-like activity was induced by bath-application of aCSF (artificial cerebrospinal fluid) mixed with N-methyl-DL-aspartate (NMA; 10 μM), serotonin (5HT; 5 μM), and dopamine (DA; 50 μm) (all purchased from Sigma-Aldrich, France).

For the elemental oscillator. Neuron N1 (corresponds to neuron L3 in Figure 1B) is connected to neuron N2 (corresponds to neuron R3 in Figure 1B) by an inhibitory synapse with a synaptic weight of −7 and a percentage dissipation of 12%. The *I*_bias_ current is equal to 7 for both neurons.

**Figure 7:**
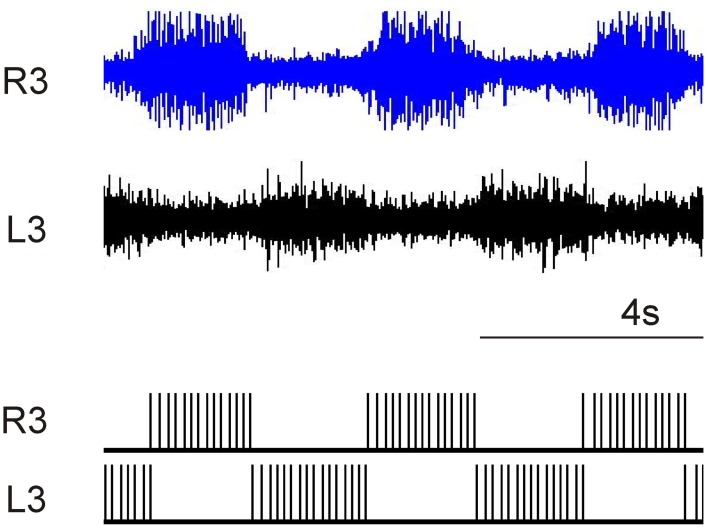
**Comparison of pharmacological *in-vitro* spinal cord with digital CPG**.

Figure 7 shows that the digital system fits the biological recordings of the newborn rat spinal cord. The period and duty cycle of the bursting activity are the same, confirming that the digital system was suitable for hybrid experiments. Instead of using pharmacological stimulation, the digital board will be used in the near future to create a hybrid experiment involving the *ex vivo* spinal cord and the digital CPGs. A closed-loop is also possible thanks to the possibility of changing the mean period of bursting activity by modifying a single parameter (τ_reg_).

## Discussion

One key step in designing a neuroprosthesis is to produce a large, resource-frugal biomimetic SNN. A biologically realistic CPG (i.e., the leech heartbeat system neural network) was implemented with a minimum resource cost in terms of neuron model, while maintaining its biomimetic activity, as shown in the Results. The first step was to model the biological leech heartbeat system using a single, segmental CPG. The next stage was to choose an efficient neuron model that required few resources for its digital implementation but remained biorealistic enough to match the behavior of biological cells. The topology and hardware implementation of a single neuron were then extended to form a neuron computation core built into a large-scale neural network: 240 CPGs on a Spartan6 FPGA board. Furthermore, the new synaptic model proposed reproduced the activity-dependent depression phenomenon, which had only previously been described in biology literature. The architecture of the entire real-time systemwas described in detail. Finally, the system was validated by several experiments comparing both elemental and segmental oscillators with biological data, and comparing the segmental oscillator with *ex vivo* rat spinal cord stimulated by pharmacological solutions.

The short-term prospect of this work is to improve the stability of the system using another neuron model. Currently our work is focused on the quartic model (Touboul, 2009), which is more stable than the Izhikevich one and also requires few resources. As described in Table 2, this system is subject to variations in duty cycle and mean period, likely to be reduced by using the new model. However, these variations also exist in biology, so it is necessary to study the actual effect of these variations in the biological system to determine whether they should be eliminated or not.

In the medium term, this system will be included in a hybrid experiment using an *ex vivo* rat spinal cord. The experiment board includes several modules, including an MEA (Micro-Electrode Array) and spike detection block, to detect and record neural activity in the spinal cord. All these modules, together with the CPG network, will be implemented in the same FPGA. Our neurophysiologist colleagues will identify the best spinal cord sites to stimulate and record bursting activity. These sites will be hybridized to the output of the artificial CPG described in this paper and, in turn, its activity will drive the various ventral root outputs of the spinal cord into full locomotor-like activity. These future experiments aim to demonstrate that hybrid artificial/biological networks provide possible solutions for rehabilitating lost central nervous system function.

Our CPG network could be also used to study the locomotion of different animals. Indeed, according to Ijspeert (2001), the locomotion activity of a salamander requires 40 CPGs, so the 240 CPGs implemented on the Spartan 6 digital board would be suitable for studying more complex locomotion. Our system will be used in a closed-loop system with different sensors and actuators.

### Conflict of interest statement

The authors declare that the research was conducted in the absence of any commercial or financial relationships that could be construed as a potential conflict of interest.
